# The Influence of the Continuous Casting Conditions on the Properties of High-Strength Two-Phase CuMg Alloys

**DOI:** 10.3390/ma13214805

**Published:** 2020-10-28

**Authors:** Paweł Strzępek, Andrzej Mamala, Małgorzata Zasadzińska, Piotr Noga, Michał Sadzikowski

**Affiliations:** Faculty of Non-Ferrous Metals, AGH University of Science and Technology, 30-059 Kraków, Poland; amamala@agh.edu.pl (A.M.); malgozas@agh.edu.pl (M.Z.); pionoga@agh.edu.pl (P.N.); msa@agh.edu.pl (M.S.)

**Keywords:** two-phase CuMg, copper alloys, SEM, continuous casting, physical and mechanical properties

## Abstract

Constant tendency toward the improvement of the material properties nowadays creates opportunities for the scientists all over the world to design and manufacture new alloys almost every day. Considering the fact that companies all over the world desire alloys with the highest values of mechanical properties often coexisting with a reasonable electrical conductivity made it necessary to develop new materials based on Cu, such as CuMg alloys. However, before such new material may be mass produced it must undergo a series of tests in order to determine the production technology, its parameters and influence on the chemical composition, microstructural properties, and both mechanical and physical properties of CuMg alloys. The research tests have shown that with the increase of the casting feed the Brinell’s hardness of each material slightly increases (by 5 HB2.5/62.5). There is little to none impact of the casting feed on the electrical conductivity, values of which are between 20.6 and 21.4 MS/m (around 40% IACS-International Annealed Copper Standard) depending on the Mg content. The conducted scanning electron microstopy (SEM) analysis has shown that the magnesium precipitations are evenly distributed among the volume of the alloy, however, a significant difference in the density and shape of the Cu + Cu_2_Mg aggregates was noticed regarding various casting feed. Static compression test proved that these alloys may be subjected to strain hardening as the hardness of the material after compression increases by approximately 40 HB2.5/62.5.

## 1. Introduction

Because of its high electrical and heat conductivity copper has a wide range of applications among many branches of industry, transportation, and everyday life. It is commonly known, that in some cases not only electrical conductivity at satisfactory level is necessary but also high mechanical properties are required which means that the use of electrolytic tough pitch (ETP) or oxygen free (OF) copper even after work hardening is not possible [1,2,3]. Various alloying additives are used in such cases and their metallurgical synthesis generates the possibilities for obtaining completely new, innovative materials such as magnesium copper. Its spectrum of applications may be very extensive especially that precipitation hardening of these alloys is possible [4], however, a certain limitation of such might be their atmospheric corrosion. However, the use of copper magnesium alloys for electrical applications for instance in the form of wires is possible with the use of specific electrolytic or galvanic coatings which would hermetically separate CuMg alloy from the atmosphere. There are many research papers accessible which concern the topic of corrosion progression and explaining the mechanisms of corrosion formation, occurring and place of origin, as well as the ways of protection from damaging outcomes with the use of, for example, the abovementioned coatings both in terms of copper alloys [5,6,7,8] and magnesium alloys [9,10,11,12]. There is only one research paper concerning copper magnesium alloys and their corrosion resistance and unfortunately it presents research results on single-phase CuMg alloys [13]. The phase diagram of CuMg alloy [14] depicts distinctly visible intermetallic Cu_2_Mg and CuMg_2_ phases (Laves phases) which were examined in [15,16] by Schubert et al. and Lieser et al. Additionally, Miroshnichenko et al. [17] proved the existence of metastable intermetallic Cu_3_Mg phase. Phase diagram also shows that two-phase CuMg alloys exist above 3% of wt.% of Mg and are formed with α phase (Cu) and intermetallic β phase (Cu_2_Mg), hardness of which has been tested and is proven to be H = 519 [18] and according to Mao et al. it is ductile [19].

In accessible literature there is a wide range of research on single-phase alloys with wt.% of Mg equal to 0.2% and 0.5% which are commercial alloys commonly used in nowadays industry [20]. Ito et al. [21] have focused on research on single-phase CuMg alloys with magnesium wt.% content above 1%. Authors of both abovementioned research papers have stated that through strain hardening of CuMg alloys it is possible to obtain ultimate tensile strength (UTS) of 700–800 MPa. The authors in [4], on the other hand, have conducted research on the influence of the heat treatment of single-phase CuMg2.26 alloy and have proven that with specific set of time and temperature it is possible to increase the strength of the alloys twofold. Considering definitely less available research on the two-phase CuMg alloys it may be stated that with the proper heat treatment and strain hardening it is possible to obtain the UTS value of above 1000 MPa [22]. Figueroa et al. have conducted research on CuMg alloys with 1% and 5% of wt.% of Mg with the addition of Sn designed for bearings and have proven that cold working of these alloys significantly decreases their deformability [23]. Another work treating on CuMg alloys with 0.4 wt.% of magnesium with micro additions of approximately 0.15 wt.% of Ce and Y was provided by Wang et al. where they stated that both additions increase the mechanical properties even more when hot deformed at the temperature range of 500 °C to 850 °C in comparison to alloys without these micro additions [24]. However, regardless of its extraordinary mechanical properties the authors in [13] claim that single-phase copper magnesium alloys have lower corrosion resistance in comparison to e.g., copper aluminum alloys in elevated temperatures and the authors attributed this fact to the incorporation of Cu in the MgO surface layer which might disqualify the use of this alloys in specific applications without the use of galvanic coating. Research works conducted all over the world by many various research teams have proven that magnesium copper alloys have very high strength properties with reasonable electrical conductivity and may function as a fine substitute to copper alloys with i.e., cadmium which is considered to be toxic in many countries [25]. One of these substitute uses was studied by Yuan et al. [26] where they discussed the wear behavior of CuMg alloys used in high-speed railway catenary cables or contact lines and their resistance to fretting and the authors claim that tribochemical reactions occurred on the contact surface, and the resultant of friction oxidation was mostly formed from CuO and Cu_2_O oxides. However, there are a few specific research papers on the usefulness of the alloys with wt.% of Mg over 1%. There are of course scientific papers on specific properties of higher Mg content like optical and electrical properties of thin metallic glass films in [27], however, there is a knowledge gap, which concerns the metallurgical synthesis and continuous casting of two-phase copper magnesium alloys, thus making it necessary to clarify the specific parameters of these processes which were conducted throughout this research and collectively presented in this research paper.

## 2. Experimental Procedures

### 2.1. Metallurgical Synthesis

**Figure 1:**
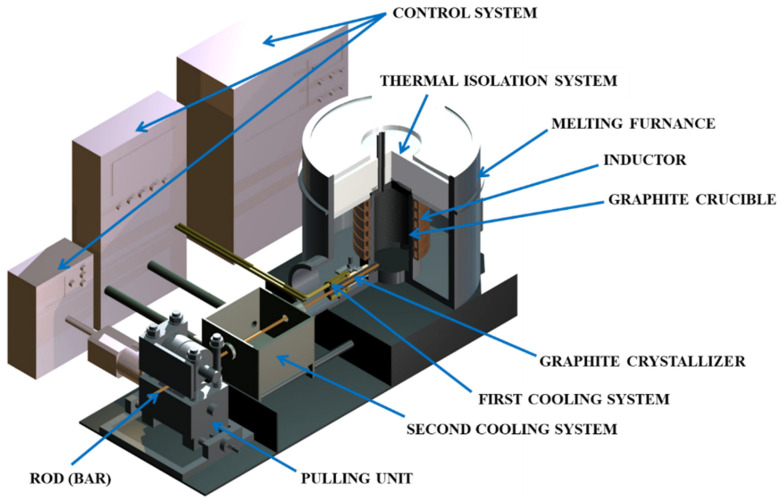
Schematics of the melting and casting furnace.

**Figure 2:**
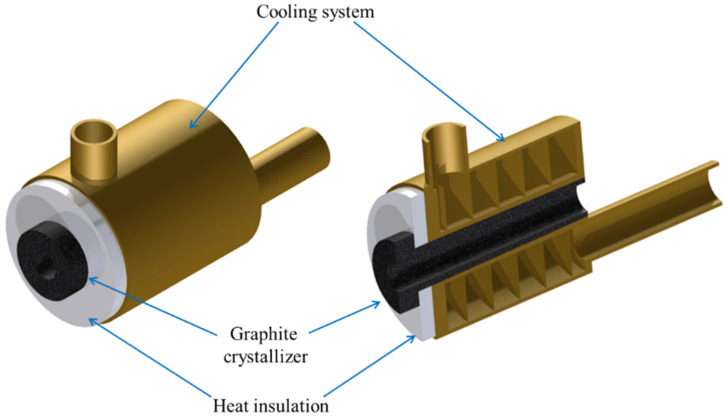
Schematics of graphite crystallizer with primary cooling system.

As part of the experimental study metallurgical synthesis of selected copper and magnesium alloys was conducted. It was established that the OF granulated copper (99.99% of Cu) and magnesium (99.9% of Mg) have been used in the metallurgical synthesis process. The melting and homogenizing process was conducted in graphite crucible of the melting and casting furnace (Figure 1) (Termetal, Piekary Śląskie, Poland) at 1250 °C and the crystallization occurred throughout the horizontal continuous casting process in the graphite crystallizer with a diameter of 14 mm with a constant primary cooling (Figure 2). The nominal power of the furnace is 20 kW and the frequency of the induction coil is 3 kHz. Because of the stable cooling conditions and unchanging temperature of the melted metal it was possible to determine the influence of the casting parameters on the selected properties of two-phase CuMg alloys. The alloys with 4.5% and 5% of wt.% of Mg casted with fixed standstill of 2 s and varied feed of 2 mm and 4 mm were evaluated. The continuous casting parameters along with the selected alloys’ compositions (at the top of the table) and the actual measured chemical compositions (at the bottom of the table) and measured density are presented in Table 1.

### 2.2. Electrical Conductivity and Hardness Measurements

The obtained cast rods were cut into samples with 10-mm thickness in as-cast state with no additional heat treatment or strain hardening which subsequently were subjected to the electrical conductivity test with the use of SigmaTest 2.069 (Forester Instruments Inc., Pittsburgh, PA, USA) which is an eddy current instrument that measures the electrical conductivity of nonferrous metals in MS/m. The measurements were conducted 24 h after the end of the continuous casting during which the samples were put in the ambient temperature in order to stabilize their thermal state. Each sample was tested with the frequency of 60 KHz. The alloys were also subjected to the Brinells hardness test with the use of Nexus3001 testing machine (Innovatest Europe BV, Maastricht, The Netherlands) with 62.5 kgf (approximately 613 N) and 10 s of indenting time. For each chemical composition and continuous casting parameters 3 different samples were selected and 15 measurements both of electrical conductivity and hardness were conducted on each. Indentations in the Brinell’s hardness test were performed with 7 at the axis of the samples and 4 on each side of the axis. Afterwards the mean value and standard deviation of each was calculated.

### 2.3. SEM and XRD Observations

Additionally, microstructure analysis using scanning electron microscopy (SEM) (Hitachi Ltd., Tokyo, Japan) using backscatter electrons was conducted with various magnifications. Along with the 5000 times magnification the chemical composition analysis and magnesium (Cu + Cu_2_Mg phase) distribution in copper matrix using energy-dispersive X-ray spectroscopy (EDX) (Hitachi Ltd., Tokyo, Japan) was conducted. Research on phase composition analysis has been performed at the ambient temperature using Rigaku MiniFlex II apparatus (Rigaku Corporation, Tokyo, Japan) which with the use of X-ray phase analysis method provided X-ray diffractions patters (XRD) (Rigaku Corporation, Tokyo, Japan). Research was conducted on alloy powder and the diffraction spectra was obtained at the 2Θ angle of between 20° and 80° with copper radiation λCu Kα = 1.5418 Å.

### 2.4. Compression Tests

Additional samples in the as-cast state with 6 mm height and 4 mm in diameter (1.5:1 relation) were subjected to 50% deformation in the static compression test and afterwards hardness and electrical conductivity tests were conducted analogically to pre-deformation samples in order to determine the strain hardening mechanisms.

## 3. Results and Discussion

### 3.1. Electrical Conductivity and Hardness Measurements

**Figure 3:**
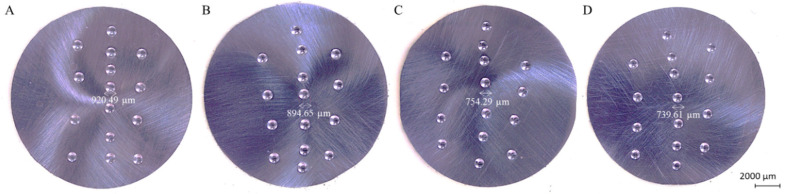
Exemplary pictures of indentations obtained in Brinell’s hardness tests; (**A**) CuMg4.5 sample casted with 2 mm feed; (**B**) CuMg4.5 sample casted with 4 mm feed, (**C**) CuMg5 samples casted with 2 mm feed, (**D**) CuMg5 samples casted with 4 mm feed.

**Figure 4:**
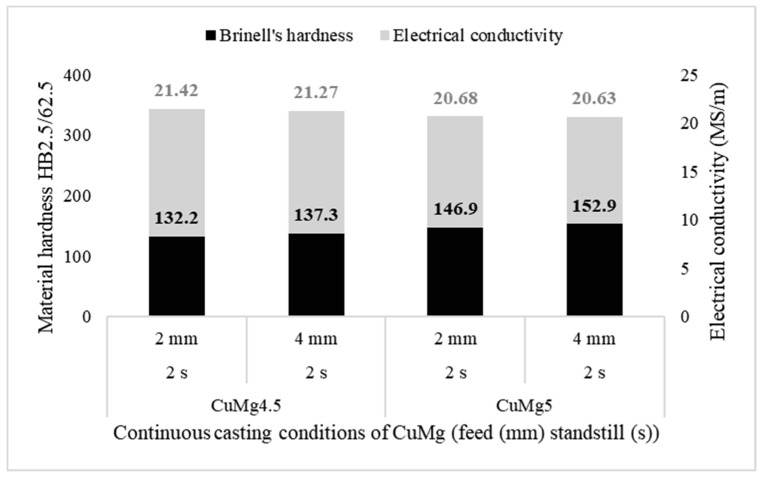
Mean values of electrical conductivity (grey columns) and Brinell’s hardness (black columns) of each of the tested samples.

Electrical and mechanical properties analysis was conducted based on the electrical conductivity research and the Brinell’s hardness test conducted afterwards. Pictures of exemplary samples after hardness tests are presented in Figure 3 with measured diameters of the middle indentations. Mean values of the obtained research results of the as-cast samples are put together in Figure 4. There were no significant macroscopic differences recorded between the conducted indentations at the cross-section of the cast rod.

**Figure 5:**
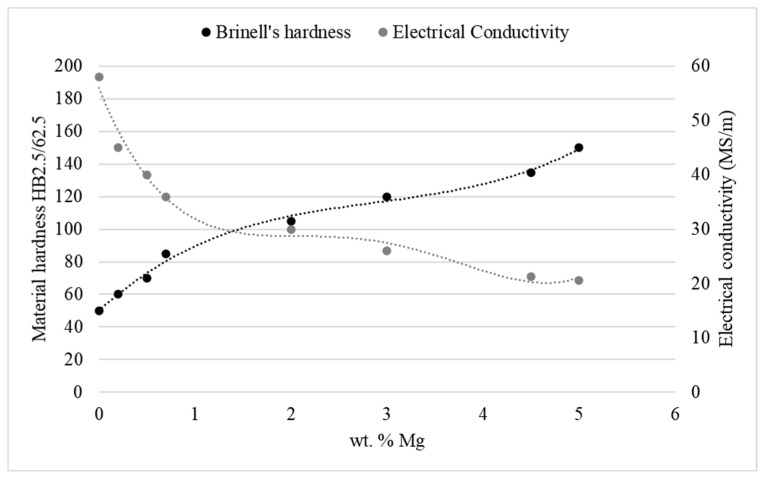
Known literature values [21,28,29] of hardness and electrical conductivity of CuMg alloys put together collectively with research results.

The calculated standard deviation values were between 0.49 and 1.16 in the case of the hardness tests and 0.24 and 0.47 in the case of the electrical conductivity measurements. Based on this it may be stated that macroscopic properties such as hardness and electrical conductivity were repeatable in the case of tested samples. It might be easily stated that as the magnesium content increases in the alloy its electrical conductivity decreases, which is described by the Nordheim’s rule and the rule of mixture. There is no significant influence of the casting feed on the obtained results. The measured values are slightly lower (up to 0.15 MS/m) in the case of the alloys casted with 4-mm feed, however, these values might be neglected because of an experimental accuracy. The increase of the magnesium wt.% from 4.5% to 5% caused the decrease of the electrical conductivity from approximately 21.42 MS/s to approximately 20.63 MS/m. It is worth noting, that according to the accessible research papers the electrical conductivity decrease is significantly higher when considering single-phase CuMg alloys i.e., from starting value of 58 MS/m (Cu) to some of the commercial CuMg alloys and others which were the focus of the scientists in [21,28,29] as presented collectively along with the research results obtained in this research paper in Figure 5. Increasing concentration of magnesium in copper solution in terms of two-phase materials caused the decrease in the electrical conductivity due to the increasing volume fraction of Cu_2_Mg intermetallic phase.

With the increase of the magnesium wt.% from 4.5% to 5% the measured Brinell’s hardness increases from approximately 135 HB to approximately 150 HB. Using the relation proposed by Tabor [30] the obtained values may be recalculated into UTS which gives values between approximately from 450 MPa to 500 MPa in the as-cast state. It may be compared with the research results of Gorsse et al. [22] who in their study determined the UTS values of the CuMg alloys to be around 230 MPa for 4.1 at.% of Mg (1 wt.%), around 370 MPa for 8.1 at.% (3.5 wt.%) and around 570 MPa for 23.1 at.% (10 wt.%). This means that the obtained values are in agreement with the values present in the accessible literature. In the case of every analyzed CuMg alloy as the casting feed increased the mean value of the hardness increased and in both cases it was approximately 5 HB2.5/62.5 which is approximately 16.5–18 MPa. It shows how in terms of CuMg alloys with no significant influence on the electrical conductivity the strength properties may increase just with various casting parameters.

### 3.2. SEM and XRD Observations

**Figure 6:**
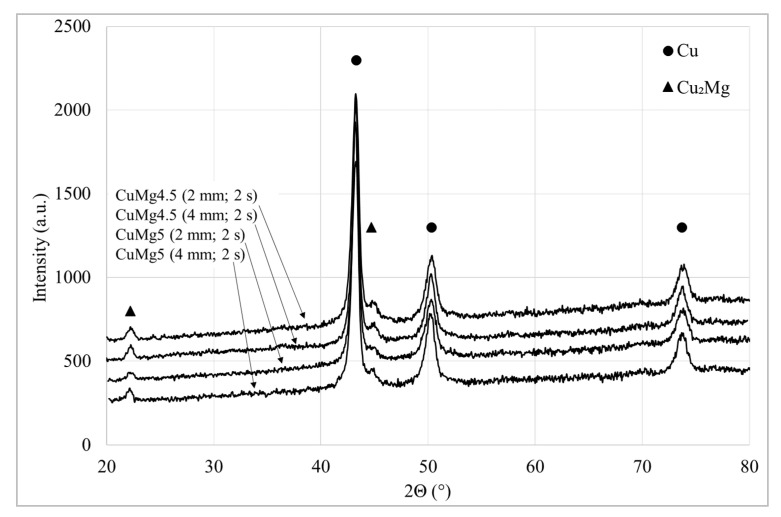
The X-ray diffraction pattern for the CuMg alloys in the as-cast state with the marked peaks.

The phase constitution of CuMg alloy powders obtained by XRD patterns is shown in Figure 6. The marked peaks clearly show the presence of Cu α phase in accordance with No. Card ICDD 00-004-0836 and Cu_2_Mg β phase in accordance with No. Card ICDD 00-058-0360.

**Figure 7:**
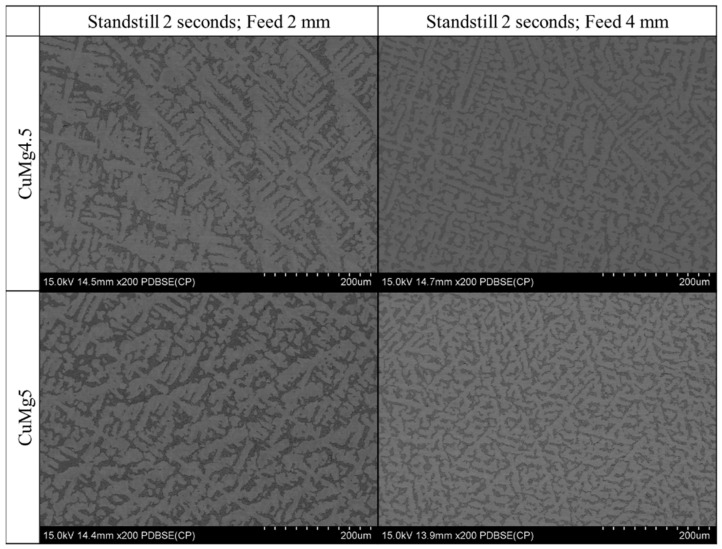
Backscatter electron SEM analysis of CuMg alloys in the as-cast state (200× magnification).

Figure 7 depicts the pictures of the microstructure of CuMg alloys obtained with backscatter electrons using SEM analysis. A clear difference is visible between CuMg4.5 and CuMg5 as there is much more Cu + Cu_2_Mg phase present in the Cu matrix as the magnesium content increases. Moreover, there is a significant difference in the packing of the Cu + Cu_2_Mg phase, as the eutectic particles form smaller but much more denser aggregates forming more grain boundary, which might explain the differences in the electrical conductivity (slightly lower with higher casting feed) and materials hardness (slightly higher with higher casting feed).

**Figure 8:**
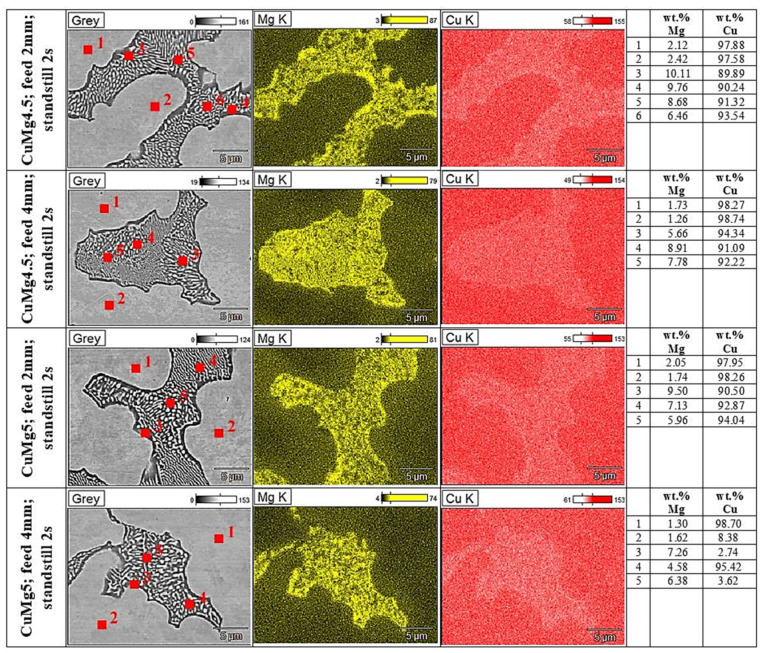
Microstructure (grey) and EDX analysis (Mg and Cu) of CuMg alloys in the as-cast state (5000× magnification).

Additional analysis conducted with SEM microscope is presented at Figure 8 along with chemical composition analysis (element mapping and point analysis). Images were obtained using backscattered electrons which based on the atomic number of the element provides specific image (the darker area for elements with lower atomic number). Chemical composition was determined using EDX detector which provides the information on the alloy matrix and precipitations based on the obtained spectrum of the alloy which allows, based on the intensity, to provide the quantitative and qualitative analysis and element mapping of the alloys. The dark areas in the pictures marked as grey show magnesium-rich phases and the bright areas show copper-rich phases.

Regardless of the magnesium content and the continuous casting parameters in all presented microstructures dendrites rich in copper occur surrounded by alternately existing Cu-rich phases (bright areas) and Mg-rich phases (dark areas). Additionally, considering the eutectic aggregates two separate morphologies of Cu + Cu_2_Mg (as shown via XRD patterns) phase may be distinguished: plate and spherical morphology which is clearly visible in the case of CuMg4.5 with 2-mm feed. Gorsse et al. [22] using die casting method have achieved similar microstructure of CuMg alloy with plate morphology of Cu + Cu_2_Mg. As the casting feed increased (with the same Mg content) the copper dendrites are smaller and the observed changes may again be correlated with the analyzed increase of hardness in the tested samples. Conducted chemical composition analysis confirmed the presence of Cu_2_Mg intermetallic phase with the mean wt.% of Mg of 9% regarding all the tested samples.

### 3.3. Compression Tests

**Figure 9:**
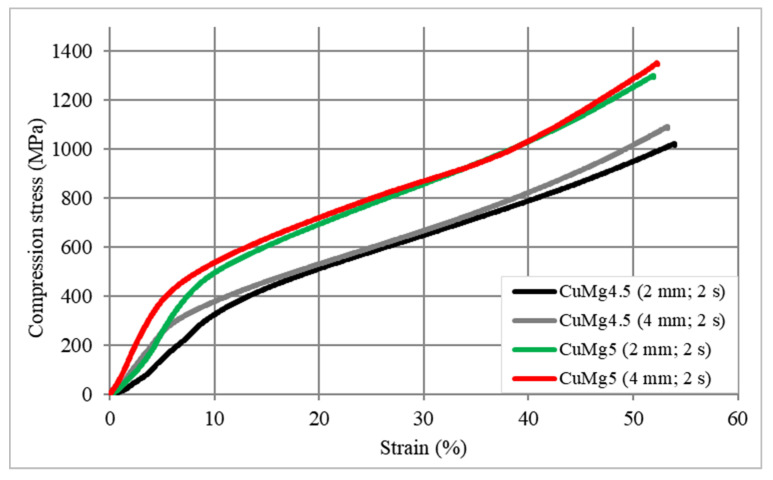
Stress–strain compression curves of CuMg alloys.

A final stage of this research was to determine the influence of the strain hardening on the tested CuMg samples in the static compression test. The exemplary stress–strain curves are presented in Figure 9 where it is clearly visible that the stress required to obtain 50% deformation is significantly higher (approximately 300 MPa difference) when considering samples with higher magnesium content which is in consistence with the hardness results of the as-cast state samples measured in this research. There is a slight difference in the compression stress–strain curves regarding the casting feed especially up to the yield strength of the tested samples, however, afterwards the curves have more or less similar course.

**Figure 10:**
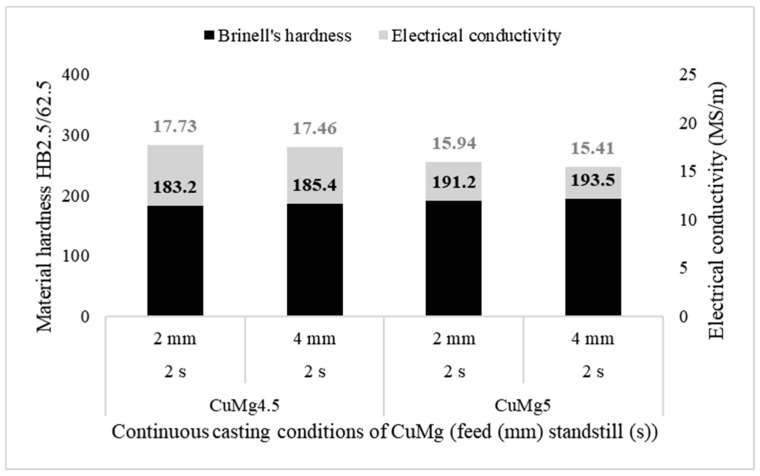
Mean values of electrical conductivity (grey columns) and Brinell’s hardness (black columns) of each of the tested samples after compression test (50% applied deformation).

Three samples taken from cast rods obtained in each of the casting parameters and chemical compositions were subjected to strain hardening in static compression test with 50% deformation and afterwards their hardness and electrical conductivity were tested again for comparison. The results of both tests are collectively presented at Figure 10.

It was found that the materials strengthens by approximately 40 HB2.5/62.5 in comparison to as-cast state which when recalculated to MPa gives UTS values of the strengthen material of approximately 605 MPa to 640 MPa. However, the electrical conductivity decreases by almost 5 MS/m which is equal to approximately 8.5% IACS. The decrease is higher regarding samples with higher casting feed, which for the as-cast samples was not that observable.

## 4. Conclusions

Taking everything into consideration, the analyzed samples did not show significant influence of the casting feed on the electrical conductivity of the two-phase CuMg alloys. Brinell’s hardness, on the other hand, and as follows material’s strength, increased as the casting feed increased by approximately 5 HB2.5/62.5 (16.5–18 MPa) which is around 4% of the base value. The increase of the casting feed caused a significant change in the density and size of the Cu + Cu_2_Mg phase aggregates, forming more grain boundaries as the casting feed increased which might be correlated with the changes in hardness of the material. XRD patterns proved the presence of Cu_2_Mg intermetallic phase and EDX analysis confirmed its presence with the mean wt% of Mg of 9%. The applied force during the compression test proved that the strain hardening of copper magnesium alloys was possible with quite significant increase of the hardness by approximately 40 HB2.5/62.5 which is around 130 MPa, however, at the same time the applied stress lowered electrical conductivity by around 5 MS/m.

## Figures and Tables

**Table 1:** Continuous casting parameters and chemical compositions of the obtained alloys.

Selected Properties and Parameters	CuMg4.5		CuMg5	
Density (g/cm^3^)	7.93		7.80	
Continuous casting parameters	Cooling medium velocity			
	Primary; Secondary			
	0.4 L/min; 0.1 L/min			
	Cooling medium temperature			
	In; Out			
	10 °C; 35–40 °C			
	Liquid metal temperature			
	1250 °C			
	Cast rod temperature			
	190 °C–220 °C			
	Standstill			
	2 s			
	Feed			
	2 mm	4 mm	2 mm	4 mm
**Element**	**wt.%**			
Mg	4.4981	4.4831	5.0073	4.9922
Cu	95.4899	95.5018	94.9815	95.0008
Ag	0.0005	00.0008	0.0007	0.0005
Zn	0.00097	0.00149	0.00164	0.00073
Pb	0.00193	0.00185	0.00207	0.00123
Fe	0.00364	0.00379	0.00237	0.00223
Ni	0.00133	0.00116	0.00106	0.00101
Sn	0.00284	0.0047	0.00271	0.00071
Si	0.0003	0.0008	0.0002	0.0001
Bi	0.0005	0.0005	0.0005	0.0005
